# Heterologous Humoral Response against H5N1, H7N3, and H9N2 Avian Influenza Viruses after Seasonal Vaccination in a European Elderly Population

**DOI:** 10.3390/vaccines5030017

**Published:** 2017-07-17

**Authors:** Ivan Sanz, Silvia Rojo, Sonia Tamames, José María Eiros, Raúl Ortiz de Lejarazu

**Affiliations:** 1 Valladolid National Influenza Centre, Avenida Ramón y Cajal s/n, 47005 Valladolid, Spain; srojor@saludcastillayleon.es (S.R.); jmeirosbouza@gmail.com (J.M.E.); rortizdelejarazu@saludcastillayleon.es (R.O.d.L.); 2 Microbiology Service, Hospital Clínico Universitario de Valladolid, Avenida Ramón y Cajal s/n, 47005 Valladolid, Spain; 3 Consejería de Sanidad, Junta de Castilla y León, Paseo de Zorrilla 1, 47007 Valladolid, Spain; TamGomSo@jcyl.es; 4 Microbiology Service, Hospital Universitario Río Hortega, Calle Dulzaina 2, 47012 Valladolid, Spain

**Keywords:** seasonal influenza vaccination, avian influenza viruses, cross-immunity

## Abstract

Avian influenza viruses are currently one of the main threats to human health in the world. Although there are some screening reports of antibodies against these viruses in humans from Western countries, most of these types of studies are conducted in poultry and market workers of Asian populations. The presence of antibodies against avian influenza viruses was evaluated in an elderly European population. An experimental study was conducted, including pre- and post-vaccine serum samples obtained from 174 elderly people vaccinated with seasonal influenza vaccines of 2006–2007, 2008–2009, 2009–2010, and 2010–2011 Northern Hemisphere vaccine campaigns. The presence of antibodies against A/H5N1, A/H7N3, and A/H9N2 avian influenza viruses were tested by using haemaglutination inhibition assays. Globally, heterotypic antibodies were found before vaccination in 2.9% of individuals against A/H5N1, 1.2% against A/H7N3, and 25.9% against A/H9N2. These pre-vaccination antibodies were present at titers ≥1/40 in 1.1% of individuals against A/H5N1, in 1.1% against H7N3, and in 0.6% against the A/H9N2 subtype. One 76 year-old male showed pre-vaccine antibodies (Abs) against those three avian influenza viruses, and another three individuals presented Abs against two different viruses. Seasonal influenza vaccination induced a significant number of heterotypic seroconversions against A/H5N1 (14.4%) and A/H9N2 (10.9%) viruses, but only one seroconversion was observed against the A/H7N3 subtype. After vaccination, four individuals showed Abs titers ≥1/40 against those three avian viruses, and 55 individuals against both A/H5N1 and A/H9N2. Seasonal vaccination is able to induce some weak heterotypic responses to viruses of avian origin in elderly individuals with no previous exposure to them. However, this response did not accomplish the European Medicament Agency criteria for influenza vaccine efficacy. The results of this study show that seasonal vaccines induce a broad response of heterotypic antibodies against avian influenza viruses, albeit at a low level.

## 1. Introduction

Seasonal influenza vaccination is conceived to protect individuals by inducing protective antibodies after annual administration. A vaccine able to induce antibodies (Abs) that can recognize all A and B influenza subtypes and lineages is under research through several approaches [1,2,3,4,5,6]. This principle is based on the heterotypic response between influenza viruses due to common parts on the structure of their most external proteins, as haemagglutinin (HA), neuraminidase (NA), and M2 [7,8,9]. Heterotypic responses are common between phylogenetically-related viruses [10,11]. This issue was widely described in a 2009 pandemic, when some authors demonstrated the existence of individuals with antibodies against the A/H1N1pdm09 subtype, even before their emergence [12,13]. Other studies have demonstrated that seasonal trivalent influenza vaccines (TIV) administered prior to 2009 induced the occurrence of antibodies able to recognize the pandemic subtype [12]. However, there is a lack of information about how TIV can induce heterotypic responses against other non-seasonal influenza viruses, such as avian influenza viruses (AIV). This issue is especially relevant in the current situation in which important avian influenza outbreaks are taking place.

AIV have been causing infections and laboratory-confirmed deaths in humans since before 1994, namely in Southeast Asian and Middle Eastern countries [14,15]. Most of the AIV infections in humans are transmitted by wild birds and poultry, but human-to-human transmission has been also described [16]. The emergence of new AIV in recent years has been constant. Surveillance conducted by World Health Organization (WHO) during 2017 is focused on four different potential pandemic AIV (A/H5N1, A/H5N6, A/H7N9, A/H9N2) [14]. The presence of those AIV subtypes is not exclusive to countries that have been traditionally affected. It has been documented that other AIV outbreaks in birds has occurred in others areas, such as Europe [17,18,19]. During 2016 and 2017, several outbreaks caused by the A/H5N8 subtype occurred in different European countries in wild birds and poultry [20,21,22,23,24]. During January of 2017, the first two cases of A/H5N8 infection in Spain were detected, causing the death of two wild geese in a lagoon of the northern region of Castile and Leon [25]. Despite that infections caused by A/H5N8 have only been documented in birds and the current risk level is low, the WHO does not exclude the appearance of sporadic human cases in the future [26].

AIV show higher lethality rates in humans than seasonal influenza viruses (SIV), ranging from 30–50% of infections [27,28]. The number of available vaccines for humans against those viruses is limited. Putting together the emergence risk of those viruses and the lack of specific vaccines for AIV, it is important to explore how seasonal vaccines could help to immunize different human populations against AIV. The aim of this study is to analyze the presence of pre-vaccine Abs against A/H5N1, A/H7N3, and A/H9N2 subtypes in a Spanish elderly population, and also to describe the heterologous humoral response after TIV immunization.

## 2. Materials and Methods

### 2.1. Patient Recruitment

A retrospective observational study was designed, obtaining pre- and post-vaccination serum samples from 174 individuals ≥65 years of age during the 2006–2007 influenza vaccine campaign (IVC) (*n*_1_ = 45), 2008–2009 IVC (*n*_2_ = 43), 2009–2010 IVC (*n*_3_ = 43), and 2010–2011 IVC (*n*_4_ = 43). Serum samples from each vaccine campaign cohort (VCC) were obtained by the clinicians of the Influenza Surveillance Network of Castile and Leon, Spain. These samples were analyzed at Valladolid National Influenza Centre for this work. Sampling of pre-vaccine serum was performed immediately before TIV administration, and post-vaccine serum sampling was performed at least 28 days after vaccination to ensure the correct immunization. Informed consent was obtained and the recruitment of the patients was done following the Spanish Organic Law for Data Protection, patient’s rights and obligations for clinical documents (BOE no. 298 of 14 December 1999, Law 41/2002). This research was performed following the Declaration of Helsinki.

### 2.2. Avian Influenza Viruses Analyzed

The analysis of the presence of Abs against AIV were performed against three different avian subtypes that have been responsible for the main human outbreaks since 1994: A/H5N1 (A/Vietnam/1194/2004), A/H7N3 (A/Canada/rv504/2004), and A/H9N2 (A/Hong Kong/1074/1997). These viruses were supplied by the NIBSC (National Institute for Biological Standards and Controls; London, UK). The strains used were isolated from avian influenza infections in humans, and then propagated at the origin in chicken embryos and partially purified, concentrated, and inactivated for use in BSL-II safety facilities by the NIBSC.

### 2.3. Composition of Seasonal Influenza Vaccines Administered

Immunization of the elderly people included in the study was performed using seasonal influenza vaccines. The TIV administered to the four different VCC had the WHO-recommended composition for the Northern Hemisphere in each IVC [29,30,31,32] (Table 1). The A/H1N1pdm09 subtype was not present during IVCs from 2006 to 2009, nor was the A/H1N1 subtype included in the 2010–2011 IVC. The A/H3N2 subtype was included in all IVCs.

### 2.4. Haemagglutination Inhibition Assay

The analysis of the presence of haemagglutinant Abs in pre- and post-vaccine serum samples were performed by means of a haemagglutination inhibition assay (HAI). This assay is one of the most recommended methodologies by WHO and the Influenza Surveillance Network for influenza surveillance and the analysis of Abs against influenza viruses [33,34]. First, the three AIV were tittered using 0.75% hen diluted erythrocytes, and the viral titer was standardized at 4 haemagglutination units (4HU). Serum samples were treated with RDE (Receptor Destroying Enzyme; Denka Seiken, Tokyo, Japan) before HAI for removing unspecific inhibitors present in the blood. HAI assay was performed in 96-V-well plates. In this plate, double-serial-dilutions of serum samples until a 1/1280 titration value was performed. Samples were incubated with 50 µL of 4HU diluted virus for 30 min at room temperature. A total amount of 50 µL of 0.75% hen erythrocytes were added and the plates were read after 30 min of incubation at room temperature. Pre- and post-vaccination titers were included in a database for their study. The hen erythrocytes were previously tested using specific antisera against AIV supplied by NIBSC. Microneutralization assay was not used because the biosafety requirements, biological containment, and security level required for research with High Pathogenic Avian Influenza viruses was not available in the laboratory.

### 2.5. Phylogenetic Analysis

A phylogenetic analysis of the type A HA gene of AIV and SIV was performed to study the phylogenetic relationships between those viruses. In that analysis, all AIV strains included in the study were included (Influenza Research Database Accession Number [IRDAN]: A/H5N1-EF541402; A/H7N3-CY015006; A/H9N2-GU053179). Also, the SIV strains were included in the TIV of all IVCs analyzed (A/H1N1 IRDAN: A/New Caledonia/20/1999-AY289929; A/Brisbane/59/2007-CY163776) (A/H3N2 IRDAN: A/Wisconsin/67/2005-CY163912; A/Brisbane/10/2007-EU199366; A/Perth/16/2009-KM821346) (A/H1N1pdm09 IRDAN: CY121680). Additionally, in this analysis the strain A/South Carolina/1/1918 of the 1918 Spanish flu (IRDAN: AF117241) and A/Albany/22/1957 of the A/H2N2 subtype (IRDAN: CY022013) were included for a wider visualization of the phylogenetic relationships of influenza A viruses that have affected humans during the last and the current centuries. Since HIA is only able to identify Abs that exclusively recognize the globular head of haemagglutinin and do not identify those that are stalk-specific [35], genetic analysis was only performed for the HA1 subunit. HA1 DNA sequences of all viruses analyzed were aligned using the ClustalW algorithm of BioEdit 7.2.3. The phylogenetic analysis was performed using MEGA 5.2 software (MegaSoftware, Tempe, AZ, USA), and the best nucleotide substitution model was predicted by the Best-Fit tool. The general time reversible model, with gamma-distributed rates, obtained the highest Bayesian information criterion score. Reproducibility of the phylogenetic tree was guaranteed by a bootstrap analysis of 1000 replications. A genetic similarity matrix for the HA1 subunit was also constructed using the maximum composite likelihood algorithm. The genetic similarity matrix was expressed as a percentage of genetic homology (% of similarity/100).

### 2.6. Statistical Analysis

The results obtained were statistical analyzed using the classical serological European Medicament Agency (EMA) criteria for the evaluation of influenza vaccine efficacy [36]. Those criteria established that populations ≥60 years of age must show a seroprotection rate ≥60% (SPR), seroconversion rate ≥30% (SCR), and an increase between pre- and post-vaccination geometric mean titers (GMTs) ≥2.0. The geometric mean increase was calculated as the rate between post- and pre-vaccination GMTs. The negative results obtained in HAI were assumed as half of the detection value (1/10) for the calculation of the GMTs [37]. Although there is a lack of consensus assuming a specific protective titer for AIV, a titer ≥1/40 was considered as protective in this work [38]. Seroconversion was defined as an increase of at least four-fold titers between pre- and post-vaccine serum samples. Additionally, seroconversion was considered a negative titer that reaches ≥1/40. Different statistical parametric and non-parametric methods were used, such as the Bonferroni test and McNemar test, by means of SPSS V20 (IBM, Armonk, NY, USA). Statistical significance was taken at the *p* < 0.05 value.

## 3. Results

### 3.1. Population Characteristics

Mean age of the groups recruited for this study was 74.7 years (Confidence Interval (CI) 95%: 72.9–77.0) during the 2006–2007 VCC, 79.2 years during the 2008–2009 VCC (CI 95%: 76.7–81.4), 74.8 years in the 2009–2010 VCC (CI 95%: 72.7–76.8), and 75.3 years in the 2010–2011 VCC (CI 95%: 72.8–77.7). Significant differences between the mean age of the different VCCs were observed, being higher in the 2008–2009 VCC (Bonferroni = 3.678; *p* = 0.013). Men comprised 64.4% (*n*_1_ = 29) of those individuals recruited during the 2006–2007 VCC, 46.5% (*n*_2_ = 20) in the 2008–2009 VCC, 62.8% (*n*_3_ = 27) in the 2009–2010 VCC, and 55.8% (*n*_4_ = 24) in the 2010–2011 VCC. All post-vaccine serum samples obtained were sampled at least 28 days after vaccination.

### 3.2. Presence of Pre- and Post-Vaccine Heterotypic Abs against AIV

Globally, 47 individuals (27.0%) showed pre-vaccine heterotypic antibodies against any of the AIV tested. After seasonal influenza vaccination, this figure increased to 134 individuals (77.0%). Interestingly, one 76-year-old male showed heterotypic Abs against those three AIV previous to seasonal vaccination, one 85-year-old male showed Abs against A/H7N3 and A/H9N2, and a 67-year-old male and 80-year-old woman showed Abs against both A/H5N1 and A/H9N2 subtypes. After seasonal vaccination, four (2.3%) individuals presented heterotypic Abs against those three AIV and 55 (31.6%) individuals against both A/H5N1 and A/H9N2. Heterotypic seroconversion induced by seasonal vaccination was assessed in 14.4% of individuals for A/H5N1, 0.6% for the A/H7N3 subtype, and 10.9% for the A/H9N2 subtype.

The presence of pre-vaccine Abs against A/H5N1 virus was detected in five individuals (2.9%) and was distributed among the VCCs as follows: one in the 2006–2007 VCC (2.2%), one in the 2009–2010 VCC (2.3%), and three in the 2010–2011 VCC (7.0%). Pre-vaccine Abs were observed in two individuals (1.2%) against the A/H7N3 virus, distributed as follows: one in the 2006–2007 VCC (2.2%) and one in the 2009–2010 VCC (2.3%). Pre-vaccine Abs against the A/H9N2 subtype were observed in 45 individuals (25.9%), distributed as follows: 11 in the 2006–2007 VCC (24.4%), 10 in the 2008–2009 VCC (23.3%), 14 in the 2009–2010 VCC (32.6%), and 10 in 2010–2011 VCC (23.3%). Most of the serum samples that showed pre-vaccine Abs in this study had titers ranging from 1/10 to 1/20. Pre-vaccine sera with protective titers (≥1/40) were observed in two individuals (1.1%) against the A/H5N1 subtype, in one (0.6%) person against the A/H7N3 subtype, and in one person against the A/H9N2 (0.6%) subtype.

### 3.3. Heterotypic Abs Response Induced by Influenza Seasonal Vaccination

The number of heterotypic seroconversions induced by seasonal influenza vaccination and the seroconversion rate in each VCC are described in Table 2. Heterotypic seroconversion was highly significant against A/H5N1 in the 2006–2007 and 2010–2011 VCCs and in the 2006–2007 VCC against A/H9N2 (McNemar; *p* < 0.01). Seroconversion was only observed in one individual against A/H7N3 in the 2006–2007 VCC.

Pre- and post-vaccine GMTs against each AIV in the VCCs analyzed are shown in Table 3. Pre-vaccine GMTs were similar in all VCCs for the A/H5N1 and A/H7N3 subtypes, ranging from 4.9 to 5.3. Pre-vaccine GMTs for the A/H9N2 subtype were slightly higher than GMT values against the others AIV in all VCCs, ranging from 5.9 (2010–2011 VCC) to 6.7 (2009–2010 VCC). The highest post-vaccine GMTs against the A/H5N1 subtype were observed in the 2010–2011 VCC (14.0) followed by the 2006–2007 VCC (11.3). Post-vaccine GMTs against the A/H7N3 subtype were similar to the pre-vaccine GMTs, ranging from 4.9 to 5.3. The highest post-vaccine GMTs against A/H9N2 were observed in the 2006–2007 VCC (19.7), while the other VCCs showed values ranging from 8.2 to 9.2.

The analysis of the efficacy of the humoral heterotypic response after TIV administration was assessed by applying classical EMA criteria for people ≥60 years old. SPR and SCR were lower than 60% and 30%, respectively, in all VCCs analyzed against the A/H5N1 subtype (Table 4). The SCR reached in 2010–2011 VCC against this subtype was 27.9%, nearly at the SCR cut-off for EMA criteria. The GMT increase was higher than 2.0 in 2006–07 and 2010–11 VCCs against the A/H5N1 subtype. Neither SPR nor SCR were higher than 60% and 30%, respectively, against the A/H9N2 subtype in any of the VCCs analyzed. The GMT increase was higher than 2.0 against A/H9N2 only in 2006–07 VCC (3.2). For the A/H7N3 subtype, none of the classical EMA criteria were reached.

### 3.4. Phylogenetic Analysis of AIV and SIV

**Figure 1:**
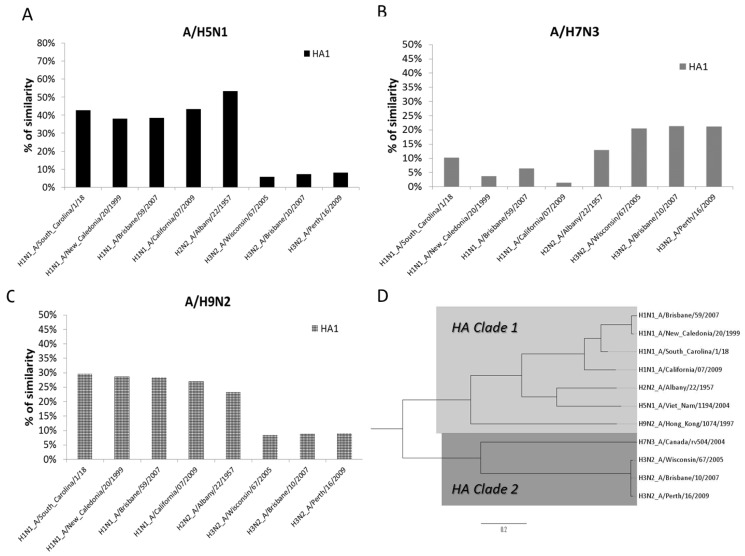
Percentage of the similarity between HA1 subunits of avian influenza viruses analyzed and those included in seasonal vaccines, as well as other influenza viruses of epidemiological interest. (**A**) Percentages of similarity against A/H5N1 avian influenza virus; (**B**) percentages of similarity against A/H7N3 avian influenza virus; (**C**) percentages of similarity against A/H9N2 avian influenza virus; and (**D**) the phylogenetic tree of the whole HA gene DNA sequence of avian influenza viruses, seasonal A influenza viruses, and other viruses of epidemiological interest.

The percentages of genetic similarity (% of similarity/100) between the different AIV and SIV are described in Table 5. Mean genetic similarity between the HA1 subunit of all of the A/H1N1 and A/H3N2 influenza strains included in TIV against the A/H5N1 subtype was 0.383 (38.3%) (CI 95%; 0.380–0.385) and 0.078 (7.8%) (CI 95%; 0.073–0.082), respectively, while against the A/H7N3 subtype it was 0.050 (5.0%) (CI 95%; 0.037–0.063) and 0.212 (21.2%) (CI 95%; 0.211–0.212), respectively, and against the A/H9N2 subtype it was 0.285 (28.5%) (CI 95%; 0.284–0.285) and 0.187 (18.7%) (CI 95%; 0.089–0.284), respectively. The analysis between the A/H2N2 subtype and the AIV showed a genetic similarity of 0.532 (53.2%) against the A/H5N1 subtype, 0.128 (12.8%) against the A/H7N3 subtype, and 0.233 (23.3%) against the A/H9N2 subtype. The phylogenetic tree constructed by using the HA sequences of all viruses analyzed is shown in Figure 1. This phylogenetic tree was constructed by using the whole HA gene (HA1 and HA2 subunits) DNA sequence. Thus, the phylogenetic tree differenced those influenza viruses analyzed in main Clades; Clade 1 includes A/H1N1, A/H1N1pdm09, A/H2N2, A/H5N1, and A/H9N2 viruses, and Clade 2 includes A/H3N2 and A/H7N3 viruses.

## 4. Discussion

Our study shows, for the first time, the results of humoral heterotypic responses against avian influenza viruses in an elderly population after seasonal influenza vaccination. Studies evaluating the presence of Abs against AIV and heterotypic responses after seasonal vaccination are scarce in Western populations. Most are performed in people with suspected infections and/or during local Western or European outbreaks in birds [39,40,41]. Many of the studies published analyze the seroprevalence of Abs against AIV in people from Southeast Asian and Middle Eastern countries, and are frequently focused in poultry workers [42,43,44,45]. The results of our study showed the presence of heterotypic Abs in 27.0% of elderly people before seasonal vaccination, and later these Abs were present in 77.0% of vaccinated people. European countries have suffered virologically-confirmed AIV outbreaks in wild birds and poultry since the last decade [18,21,22,23,24,25,46], and currently these viruses are still circulating. Thus, it is important to explore the prevalence of Abs of different European populations against AIV, as well as the utility of seasonal vaccines to trigger a heterotypic response. In a hypothetical AIV outbreak in humans, one of the most affected populations could be the elderly.

The results of our study showed the existence of Abs that can specifically recognize the globular head of HA of AIV in an elderly Spanish population. Most of these Abs were present at low titers ranging from 1/10 to 1/20. However, we observed a low percentage of elderly people that presented Abs considered protective in our work (≥1/40) against A/H5N1, A/H7N3, and A/H9N2. The existence of some individuals with protective Abs before vaccination is anecdotal, although it is interesting to note the higher number of individuals with Abs at lower titers against the A/H9N2 subtype. The elderly population is considered a risk groups for influenza vaccination, and in outbreaks of avian influenza viruses may present the most severe cases [47]. The high number of experiences against different influenza viruses through natural infections and vaccinations of this age group along their lives could have induced some degree of seroprotection against other influenza subtypes. We have checked this issue in our work and we have observed that some individuals broadly present Abs able to recognize all tested AIVs [48,49]. This is an important rationale for the latest approach of the universal influenza vaccine.

Seasonal influenza vaccination induced a low, but significant, heterotypic seroconversion against A/H5N1 and A/H9N2 subtypes in some of the VCCs analyzed, ranging from 20.0–27.9% of the people analyzed. However, heterotypic seroconversion was not observed against the A/H7N3 subtype. Our results show that TIV significantly increased the Abs titers against A/H5N1 and A/H9N2 subtypes in some vaccinated elderly people. This heterotypic seroconversion was not observed in all VCCs analyzed, and was only significant against A/H5N1 in the 2006–2007 VCC (20.0%) that was vaccinated against the A/H1N1 subtype, and in the 2010–2011 VCC (27.9%) that was vaccinated against the A/H1N1pdm09 subtype. These data suggest that cross-immunity induced by TIV administration depends on the influenza strains included in the vaccine composition. We only observed significant heterotypic seroconversion against the A/H9N2 subtype during the 2006–2007 VCC (24.4%). Interestingly, the heterotypic seroconversion observed against Clade 1 viruses was different in the elderly population vaccinated against the A/H1N1 subtype compared to those vaccinated against the A/H1N1pdm09 subtype. We observed that TIV-A/H1N1pdm09 induced a higher SCR than TIV-A/H1N1 against the A/H5N1 subtype, while TIV-A/H1N1pdm09 did not induce any heterotypic response against the A/H9N2 subtype.

The origin of the pre-vaccine Abs and heterotypic responses found in the elderly people analyzed in this work is unknown. It is improbable that these Abs have been produced by natural exposures to AIV in those people. One of the possible explanations to this issue is that these Abs have been induced by heterotypic or cross-immune reactions caused by previous infections with seasonal viruses or by repeated seasonal vaccinations. The mean age of the elderly people analyzed ranged from 74.7 to 79.2 years old, which means that this population was born before 1946, so they have been exposed to a wide number of SIV A types, most of them even to Spanish Influenza virus [50], and they probably have been vaccinated several times in their lives as a risk group. It is probable that this high number of experiences against different influenza viruses is responsible for the presence of these heterotypic responses.

The phylogenetic analysis performed in our work showed that the genetic homology between the HA1 subunit of A/H5N1 and A/H9N2 subtypes against the A/H1N1 subtype was 38.3% and 28.5%, respectively. These results show a low to moderate homology between HA1 subunits of the A/H1N1 subtype and the A/H5N1 and A/H9N2 subtypes. The methodology used in this work has a weakness that did not allow us to check the presence of Abs directed at the HA2 subunit [35]. This subunit has a higher conserved structure between different A influenza viruses and presents a lower mutation rate than HA1. The HA2 subunit is a main target for a wide number of Abs able to recognize different influenza subtypes [51]. Actually, it is the target of different research projects currently conducted for the design of a universal flu vaccine [4,5,6,52]. The heterotypic Abs observed in our work are probably globular head-specific. Although the HA1 subunit is less conserved than HA2 subunit, our results showed that it also has a potential for inducing cross-immune reactions. This could also explain the presence of heterotypic Abs in the studied population for the viruses belonging to Clade 1 [53].

The results of the phylogenetic analysis supports the idea that heterotypic responses are induced by the presence of highly-conserved epitopes in the HA1 subunit [54,55,56]. Some of these conserved epitopes have been described by other authors in the type 1, 2, 4, and 6 HA proteins [7,56]. Cross-reactions are frequent and have been observed against different influenza viruses with the same HA protein (A/H5N1 and A/H5N3) [57,58], and also between viruses from the same clade [59,60]. However, as far as we know, this is the first work that shows heterotypic seroconversion against AIV after seasonal vaccination in a European elderly population by using HA1 globular head-specific Abs. The absence of heterotypic Abs against A/H7N3 is probably caused by the low genetic similarity between this subtype and the A/H3N2 subtype. This SIV is the closest virus included in the TIV that could have naturally infected the studied population. Both A/H7N3 and A/H3N2 presented a 21.2% of homology in the HA1 subunit, which seems insufficient to allow cross-immunity between Clade 2 viruses. Nevertheless, one study demonstrates the existence of conserved epitopes between Clade 2 viruses that induced cross-reactions [61], which was not observed in our work.

The A/H2N2 subtype can be also implicated in the heterotypic responses observed in our work. The presence of Abs against this subtype was not verified due to biosafety reasons. The age of the people analyzed in our study make it probable that they have been in contact with the A/H2N2 subtype, which emerged during 1957 and circulated until 1968 [62]. The phylogenetic study showed that the genetic homology between the HA1 subunit of the A/H2N2 and A/H5N1 subtypes is 53.2%, whereas it was lower between the A/H1N1 and A/H5N1 subtypes (38.3%). These results could explain that past exposures to A/H1N1 and A/H2N2 viruses can be responsible for the presence of Abs that recognize different AIV from Clade 1, and also be responsible for the heterotypic responses after TIV administration. To verify this issue it will be necessary to study the presence of Abs in younger people that have only been in contact with A/H3N2 [62] and emergent A/H1N1 subtypes [63].

Our results also showed that homology between protein sequences from different influenza A subtypes is not the unique factor implicated in cross-reactions [64]. The presence of a moderate percentage of elderly people with pre-vaccine Abs against A/H9N2 at low titers is surprising due to the genetic similarity between A/H9N2 and A/H1N1 or A/H2N2 being lower than A/H5N1. Nevertheless, the percentage of people that presented protective titers were quite similar. On the other hand, a similar issue was observed regarding heterotypic seroconversion against A/H5N1 and A/H9N2. Seasonal vaccination induced a higher heterotypic seroconversion against A/H5N1 when people were vaccinated against A/H1N1pdm09, a subtype that presented a higher homology than A/H1N1. However, this issue was not observed against the A/H9N2 subtype, and vaccination only induced a significant heterotypic response when people were vaccinated against the A/New Caledonia/20/99 A/H1N1 strain. Both issues are difficult to explain by themselves without the study of a higher number of individuals, but probably manifests that genetic and antigenic distances are not equal. Thus, deeper proteomic research is needed to compare the genetic and antigenic differences observed.

## 5. Conclusions

The results of our study show the existence of heterotypic Abs against AIV in a population never exposed to those viruses, and an increase in those Abs after seasonal influenza vaccination. This interesting heterotypic response to vaccination was limited, and seroconversion was observed in 14.4% individuals for A/H5N1, 0.6% for A/H7N3, and 10.9% for A/H9N2, but after vaccination 77.0% of individuals showed Abs against any of AIV analyzed. A deeper research in other age groups is needed to explore this issue in other populations that have been exposed fewer times to seasonal influenza viruses compared to the elderly.

## Figures and Tables

**Table 1:** A and B influenza strains included in the seasonal influenza vaccines administered to the cohorts, analyzed following the recommended vaccine composition for the Northern Hemisphere ^1^.

Type	Subtype	Influenza Vaccine Campaigns			
		2006–2007	2008–2009	2009–2010	2010–2011
A	H1N1	A/New Caledonia/20/99	A/Brisbane/59/2007	A/Brisbane/59/2007	Not included
	H3N2	A/Wisconsin/67/2005	A/Brisbane/10/2007	A/Brisbane/10/2007	A/Victoria/201/2009
	H1N1pdm09	Not included	Not included	Not included	A/California/07/2009
B	Yamagata	Not included	B/Florida/4/2006	Not included	Not included
	Victoria	B/Malaysia/2506/2004	Not included	B/Brisbane/60/2008	B/Brisbane/60/2008

^1^ World Health Organization (WHO) vaccine recommendations.

**Table 2:** Number of seroconversions and seroconversion rate against avian influenza viruses after trivalent influenza vaccine shot in each vaccine campaign cohort.

Vaccinated Cohorts	A/H5N1		A/H7N3		A/H9N2	
	SCn ^1^	SCR ^2^	SCn	SCR	SCn	SCR
2006–2007	9	20.0	1	2.2	11	24.4
2008–2009	1	2.3	0	0.0	3	7.0
2009–2010	3	7.0	0	0.0	2	4.7
2010–2011	12	27.9	0	0.0	3	7.0

^1^ Number of seroconversions; ^2^ Seroconversion rate.

**Table 3:** Geometric mean titers and CI 95% observed against avian influenza viruses before and after trivalent influenza vaccination in each vaccine campaign cohort.

AIV ^1^	Input	Vaccinated Cohorts			
		2006–2007	2008–2009	2009–2010	2010–2011
A/H5N1					
	Pre-vaccine GMT^2^ (CI 95%)	5.3 (4.9–6.0)	4.9 (4.9–4.9)	5.2 (4.9–5.8)	5.3 (4.9–5.6)
	Post-vaccine GMT (CI 95%)	11.3 (8.4–15.9)	6.5 (5.7–7.6)	8.5 (7.0–10.5)	14.0 (10.8–18.4)
A/H7N3					
	Pre-vaccine GMT (CI 95%)	5.3 (4.9–6.0)	4.9 (4.9–4.9)	5.2 (4.9–5.5)	4.9 (4.9–4.9)
	Post-vaccine GMT (CI 95%)	5.3 (4.9–6.0)	4.9 (4.9–4.9)	5.2 (4.9–5.5)	5.3 (4.9–5.9)
A/H9N2					
	Pre-vaccine GMT (CI 95%)	6.1 (5.5–6.9)	6.0 (5.4–6.6)	6.7 (5.9–7.7)	5.9 (5.4–6.4)
	Post-vaccine GMT (CI 95%)	19.7 (14.0–29.4)	9.2 (7.6–11.2)	8.9 (7.5–10.8)	8.2 (6.9–10.0)

^1^ Avian influenza virus; ^2^ Geometric mean titers.

**Table 4:** Values of seroprotection rate, seroconversion rate, and geometric mean titer increase against avian influenza viruses after trivalent influenza vaccination in each vaccine campaign cohort.

AIV ^1^	Input	Vaccinated Cohorts			
		2006–2007	2008–2009	2009–2010	2010–2011
A/H5N1					
	SPR ^2^	20.0	2.3	9.3	27.9
	SCR ^3^	20.0	2.3	7.0	27.9
	GMT increase	2.1	1.3	1.6	2.7
A/H7N3					
	SPR	2.2	0.0	0.0	0.0
	SCR	0.0	0.0	0.0	0.0
	GMT increase	1.0	1.0	1.0	1.1
A/H9N2					
	SPR	28.9	7.0	7.0	7.0
	SCR	24.4	7.0	4.7	7.0
	GMT increase	3.2	1.5	1.3	1.4

^1^ Avian influenza virus; ^2^ Seroprotection rate; ^3^ Seroconversion rate.

**Table 5:** Genetic similarity expressed as a percentage of similarity/100 of the HA1 subunit of the haemagglutinin gene between the different avian influenza viruses analyzed and those included in seasonal vaccines, as well as other influenza viruses of epidemiological interest.

Influenza A Subtypes and Strains	A/H1N1 (A/South_Carolina/1/18)	A/H1N1pdm09 (A/California/07/2009)	A/H2N2 (A/Albany/22/1957)	A/H5N1 (A/VietNam/1194/2004)	A/H7N3 (A/Canada/rv504/2004)	A/H9N2 (A/Hong Kong/1074/1997)	A/H3N2 (A/Brisbane/10/2007)	A/H1N1 (A/Brisbane/59/2007)	A/H1N1 (A/New_Caledonia/20/1999)	A/H3N2 (A/Perth/16/2009)	A/H3N2 (A/Wisconsin/67/2005)
A/H1N1 (A/South_Carolina/1/18)	1.000										
A/H1N1pdm09 (A/California/07/2009)	0.785	1.000									
A/H2N2 (A/Albany/22/1957)	0.439	0.416	1.000								
A/H5N1 (A/VietNam/1194/2004)	0.428	0.434	0.532	1.000							
A/H7N3 (A/Canada/rv504/2004)	0.101	0.013	0.128	0.133	1.000						
A/H9N2 (A/Hong Kong/1074/1997)	0.296	0.270	0.233	0.265	0.059	1.000					
A/H3N2 (A/Brisbane/10/2007)	0.089	0.088	0.090	0.073	0.212	0.087	1.000				
A/H1N1 (A/Brisbane/59/2007)	0.802	0.680	0.395	0.385	0.063	0.284	0.013	1.000			
A/H1N1 (A/New Caledonia/20/1999)	0.815	0.689	0.399	0.380	0.037	0.285	0.013	0.969	1.000		
A/H3N2 (A/Perth/16/2009)	0.082	0.076	0.082	0.082	0.211	0.089	0.988	0.000	0.006	1.000	
A/H3N2 (A/Wisconsin/67/2005)	0.082	0.087	0.082	0.058	0.204	0.083	0.989	0.010	0.011	0.983	1.000

## References

[1] Nachbagauer R., Krammer F. (2017). Universal influenza virus vaccines and therapeutic antibodies. Clin. Microbiol. Infect.

[2] Krammer F. (2017). Strategies to induce broadly protective antibody responses to viral glycoproteins. Expert Rev. Vaccines.

[3] Impagliazzo A., Milder F., Kuipers H., Wagner M.V., Zhu X., Hoffman R.M.B., van Meersbergen R., Huizingh J., Wanningen P., Verspuij J. (2015). A stable trimeric influenza hemagglutinin stem as a broadly protective immunogen. Science.

[4] Chen C.-J., Ermler M.E., Tan G.S., Krammer F., Palese P., Hai R. (2016). Influenza A Viruses Expressing Intra- or Inter-group Chimeric Hemagglutinins. J. Virol..

[5] Ermler M.E., Kirkpatrick E., Sun W., Hai R., Amanat F., Chromikova V., Palese P., Krammer F. (2017). Chimeric Hemagglutinin Constructs Induce Broad Protection against Influenza B Virus Challenge in the Mouse Model. J. Virol..

[6] Valkenburg S.A., Mallajosyula V.V.A., Li O.T.W., Chin A.W.H., Carnell G., Temperton N., Varadarajan R., Poon L.L.M. (2016). Stalking influenza by vaccination with pre-fusion headless HA mini-stem. Sci. Rep..

[7] Wyrzucki A., Dreyfus C., Kohler I., Steck M., Wilson I.A., Hangartner L. (2014). Alternative recognition of the conserved stem epitope in influenza A virus hemagglutinin by a VH3-30-encoded heterosubtypic antibody. J. Virol..

[8] Feery B.J., Evered M.G., Hayes K. (1978). Homologous and heterologous antibody responses to subunit influenza virus vaccine. J. Hyg..

[9] Kolpe A., Schepens B., Fiers W., Saelens X. (2017). M2-based influenza vaccines: Recent advances and clinical potential. Expert Rev. Vaccines.

[10] Epstein S.L., Price G.E. (2010). Cross-protective immunity to influenza A viruses. Expert Rev. Vaccines.

[11] Lara-Ramírez E.E., Segura-Cabrera A., Salazar M.I., Rodríguez-Pérez M.A., Guo X. (2013). Large scale genome analysis shows that the epitopes for broadly cross-reactive antibodies are predominant in the pandemic 2009 influenza virus A H1N1 strain. Viruses.

[12] Hancock K., Veguilla V., Lu X., Zhong W., Butler E.N., Sun H., Liu F., Dong L., DeVos J.R., Gargiullo P.M. (2009). Cross-reactive antibody responses to the 2009 pandemic H1N1 influenza virus. N. Engl. J. Med..

[13] Ikonen N., Strengell M., Kinnunen L., Osterlund P., Pirhonen J., Broman M., Davidkin I., Ziegler T., Julkunen I. (2010). High frequency of cross-reacting antibodies against 2009 pandemic influenza A(H1N1) virus among the elderly in Finland. Euro Surveill..

[14] WHO Avian Influenza Weekly Update Number 569 2017. http://www.wpro.who.int/emerging_diseases/ai_weekly_569_wpro_20170127_final.pdf.

[15] Pu J., Wang S., Yin Y., Zhang G., Carter R.A., Wang J., Xu G., Sun H., Wang M., Wen C. (2015). Evolution of the H9N2 influenza genotype that facilitated the genesis of the novel H7N9 virus. Proc. Natl. Acad. Sci. USA.

[16] Peiris J.S.M., de Jong M.D., Guan Y. (2007). Avian Influenza Virus (H5N1): A Threat to Human Health. Clin. Microbiol. Rev..

[17] Iglesias I., Perez A.M., Sánchez-Vizcaíno J.M., Muñoz M.J., Martínez M., de la Torre A. (2011). Reproductive ratio for the local spread of highly pathogenic avian influenza in wild bird populations of Europe, 2005–2008. Epidemiol. Infect..

[18] Iglesias I., Martínez M., Muñoz M.J., de la Torre A., Sánchez-Vizcaíno J.M. (2010). First case of highly pathogenic avian influenza in poultry in Spain. Transbound. Emerg. Dis..

[19] Hesterberg U., Harris K., Stroud D., Guberti V., Busani L., Pittman M., Piazza V., Cook A., Brown I. (2009). Avian influenza surveillance in wild birds in the European Union in 2006. Influenza Other Respir. Viruses.

[20] (2016). Call for vigilance as H5N8 avian influenza confirmed in Lincolnshire. Vet. Rec..

[21] Globig A., Starick E., Homeier T., Pohlmann A., Grund C., Wolf P., Zimmermann A., Wolf C., Heim D., Schlößer H. (2016). Epidemiological and Molecular Analysis of an Outbreak of Highly Pathogenic Avian Influenza H5N8 clade 2.3.4.4 in a German Zoo: Effective Disease Control with Minimal Culling. Transbound. Emerg. Dis..

[22] Hall S., Voas S., Glossop C., Huey R. (2016). Heightened risk of H5N8 highly pathogenic avian influenza. Vet. Rec..

[23] Adlhoch C., Brown I.H., Angelova S.G., Bálint Á., Bouwstra R., Buda S., Castrucci M.R., Dabrera G., Dán Á., Grund C. (2016). Highly pathogenic avian influenza A(H5N8) outbreaks: Protection and management of exposed people in Europe, 2014/15 and 2016. Eurosurveillance..

[24] Lee D.-H., Sharshov K., Swayne D. E., Kurskaya O., Sobolev I., Kabilov M., Alekseev A., Irza V., Shestopalov A. (2017). Novel Reassortant Clade 2.3.4.4 Avian Influenza A(H5N8) Virus in Wild Aquatic Birds, Russia, 2016. Emerg. Infect. Dis..

[25] FAO (2017). H5N8 HPAI Global situation update [Internet]. http://www.fao.org/AG/AGAINFO/PROGRAMMES/EN/empres/H5N8/situation_update.htmal#.

[26] WHO (2016). Assessment of risk associated with influenza A(H5N8) virus [Internet]. http://who.int/influenza/human_animal_interface/avian_influenza/riskassessment_AH5N8_201611/en/.

[27] WHO (2015). Avian Influenza Weekly Update Number 509 [Internet]. http://www.wpro.who.int/emerging_diseases/AvianInfluenza/en/.

[28] Su S., Bi Y., Wong G., Gray G.C., Gao G.F., Li S. (2015). Epidemiology, Evolution, and Recent Outbreaks of Avian Influenza Virus in China. J. Virol..

[29] WHO (2006). Recommendations for Influenza Vaccine Composition: Northern Hemisphere: 2006–2007 [Internet]. http://www.who.int/entity/influenza/vaccines/2007northreport.pdf.

[30] WHO (2008). Recommended Composition of Influenza Virus Vaccines for Use in the 2008–2009 Influenza Season [Internet]. http://www.who.int/entity/influenza/vaccines/recommended_compositionFeb08FullReport.pdf.

[31] WHO (2009). Recommended Composition of Influenza Virus Vaccines for Use in the 2009–2010 Influenza Sason [Internet]. http://www.who.int/entity/influenza/vaccines/200902_recommendation.pdf.

[32] WHO (2010). Recommended Viruses for Influenza Vaccines for Use in the 2010–2011 Northern Hemisphere Influenza Season [Internet]. http://www.who.int/entity/influenza/vaccines/virus/recommendations/201002_Recommendation.pdf.

[33] WHO (2007). Recommendations and Laboratory Procedures for Detection of Avian Influenza A(H5N1) Virus in Specimens from Suspected Human Cases [Internet]. http://www.who.int/influenza/resources/documents/RecAIlabtestsAug07.pdf.

[34] WHO Global Influenza, Surveillance Network Manual for the laboratory diagnosis and virological surveillance of influenza 2011. http://apps.who.int/iris/bitstream/10665/44518/1/9789241548090_eng.pdf.

[35] He W., Mullarkey C.E., Miller M.S. (2015). Measuring the neutralization potency of influenza A virus hemagglutinin stalk/stem-binding antibodies in polyclonal preparations by microneutralization assay. Methods San Diego Calif..

[36] EMA (1997). Note for Guiadance on Harmonisation of Requirements for Influenza Vaccines (CPMP/BWP/214/96) [Internet]. http://www.ema-europa.eu/docs/en_GB/document_library/Scientific_guideline/2009/09/WC500003945.pdf.

[37] Zacour M., Ward B.J., Brewer A., Tang P., Boivin G., Li Y., Warhuus M., McNeil S.A., LeBlanc J.J., Hatchette T.F. (2016). Standardization of Hemagglutination Inhibition Assay for Influenza Serology Allows for High Reproducibility between Laboratories. Clin. Vaccine Immunol. CVI.

[38] Trombetta C. M., Perini D., Mather S., Temperton N., Montomoli E. (2014). Overview of Serological Techniques for Influenza Vaccine Evaluation: Past, Present and Future. Vaccines.

[39] Meijer A., Bosman A., van de Kamp E.E.H.M., Wilbrink B., Du Ry van Beest Holle M., Koopmans M. (2006). Measurement of antibodies to avian influenza virus A(H7N7) in humans by hemagglutination inhibition test. J. Virol. Methods.

[40] Gray G.C., McCarthy T., Capuano A.W., Setterquist S.F., Alavanja M.C., Lynch C.F. (2008). Evidence for avian influenza A infections among Iowa’s agricultural workers. Influenza Other Respir. Viruses.

[41] Coman A., Maftei D.N., Krueger W.S., Heil G.L., Friary J.A., Chereches R.M., Sirlincan E., Bria P., Dragnea C., Kasler I. (2013). Serological evidence for avian H9N2 influenza virus infections among Romanian agriculture workers. J. Infect. Public Health.

[42] Dung T.C., Dinh P.N., Nam V.S., Tan L.M., Hang N.L.K., Thanh L.T., Mai L.Q. (2014). Seroprevalence survey of avian influenza A(H5N1) among live poultry market workers in northern Viet Nam, 2011. Western Pac. Surveill. Response J..

[43] Huang R., Wang A.-R., Liu Z.-H., Liang W., Li X.-X., Tang Y.-J., Miao Z.-M., Chai T.-J. (2013). Seroprevalence of avian influenza H9N2 among poultry workers in Shandong Province, China. Eur. J. Clin. Microbiol. Infect. Dis..

[44] Wang X., Fang S., Lu X., Xu C., Cowling B.J., Tang X., Peng B., Wu W., He J., Tang Y. (2014). Seroprevalence to avian influenza A(H7N9) virus among poultry workers and the general population in southern China: A longitudinal study. Clin. Infect. Dis..

[45] Zhou P., Zhu W., Gu H., Fu X., Wang L., Zheng Y., He S., Ke C., Wang H., Yuan Z. (2014). Avian influenza H9N2 seroprevalence among swine farm residents in China. J. Med. Virol..

[46] (2017). Detection of H5N8 virus in wild birds “no surprise”, says Defra. Vet. Rec..

[47] ECDC Priority risk groups for Influenza vaccination. http://ecdc.europa.eu/en/healthtopics/seasonal_influenza/vaccines/Pages/influenza_vaccination.aspx#riskgroups.

[48] Russell C.J. (2011). Stalking influenza diversity with a universal antibody. N. Engl. J. Med..

[49] Corti D., Voss J., Gamblin S.J., Codoni G., Macagno A., Jarrossay D., Vachieri S.G., Pinna D., Minola A., Vanzetta F. (2011). A neutralizing antibody selected from plasma cells that binds to group 1 and group 2 influenza A hemagglutinins. Science.

[50] Taubenberger J.K., Morens D.M. (2006). 1918 Influenza: the mother of all pandemics. Emerg. Infect. Dis..

[51] Nachbagauer R., Wohlbold T.J., Hirsh A., Hai R., Sjursen H., Palese P., Cox R.J., Krammer F. (2014). Induction of broadly reactive anti-hemagglutinin stalk antibodies by an H5N1 vaccine in humans. J. Virol..

[52] Steel J., Lowen A.C., Wang T T., Yondola M., Gao Q., Haye K., García-Sastre A., Palese P. (2010). Influenza virus vaccine based on the conserved hemagglutinin stalk domain. mBio.

[53] Air G.M. (1981). Sequence relationships among the hemagglutinin genes of 12 subtypes of influenza A virus. Proc. Natl. Acad. Sci. USA.

[54] Yamashita A., Kawashita N., Kubota-Koketsu R., Inoue Y., Watanabe Y., Ibrahim M.S., Ideno S., Yunoki M., Okuno Y., Takagi T. (2010). Highly conserved sequences for human neutralization epitope on hemagglutinin of influenza A viruses H3N2, H1N1 and H5N1: Implication for human monoclonal antibody recognition. Biochem. Biophys. Res. Commun..

[55] Sui J., Hwang W.C., Perez S., Wei G., Aird D., Chen L., Santelli E., Stec B., Cadwell G., Ali M., Wan H. (2009). Structural and functional bases for broad-spectrum neutralization of avian and human influenza A viruses. Nat. Struct. Mol. Biol..

[56] Smirnov Y.A., Lipatov A.S., Gitelman A.K., Okuno Y., Van Beek R., Osterhaus A.D., Claas E.C. (1999). An epitope shared by the hemagglutinins of H1, H2, H5, and H6 subtypes of influenza A virus. Acta Virol..

[57] Stephenson I., Bugarini R., Nicholson K.G., Podda A., Wood J.M., Zambon M.C., Katz J.M. (2005). Cross-reactivity to highly pathogenic avian influenza H5N1 viruses after vaccination with nonadjuvanted and MF59-adjuvanted influenza A/Duck/Singapore/97 (H5N3) vaccine: a potential priming strategy. J. Infect. Dis..

[58] Forrest H.L., Khalenkov A.M., Govorkova E.A., Kim J.-K., Giudice G.D., Webster R.G. (2009). Single- and multiple-clade influenza A H5N1 vaccines induce cross protection in ferrets. Vaccine.

[59] Jang Y.H., Byun Y.H., Lee Y.J., Lee Y.H., Lee K.-H., Seong B.L. (2012). Cold-adapted pandemic 2009 H1N1 influenza virus live vaccine elicits cross-reactive immune responses against seasonal and H5 influenza A viruses. J. Virol..

[60] Imai K., Nakamura K., Mase M., Tsukamoto K., Imada T., Yamaguchi S. (2007). Partial protection against challenge with the highly pathogenic H5N1 influenza virus isolated in Japan in chickens infected with the H9N2 influenza virus. Arch. Virol..

[61] Ekiert D.C., Friesen R.H.E., Bhabha G., Kwaks T., Jongeneelen M., Yu W., Ophorst C., Cox F., Korse H.J.W.M., Brandenburg B., Vogels R. (2011). A Highly Conserved Neutralizing Epitope on Group 2 Influenza A Viruses. Science.

[62] Kilbourne E.D. (2006). Influenza pandemics of the 20th century. Emerg. Infect. Dis..

[63] Kozlov J.V., Gorbulev V.G., Kurmanova A.G., Bayev A.A., Shilov A.A., Zhdanov V.M. (1981). On the origin of the H1N1 (A/USSR/90/77) influenza virus. J. Gen. Virol..

[64] Peeters B., Reemers S., Dortmans J., de Vries E., de Jong M., van de Zande S., Rottier P.J.M., de Haan C.A.M. (2017). Genetic versus antigenic differences among highly pathogenic H5N1 avian influenza A viruses: Consequences for vaccine strain selection. Virology.

